# Mendelian randomization investigation identified the causal relationship between body fat indexes and the risk of bladder cancer

**DOI:** 10.7717/peerj.14739

**Published:** 2023-01-20

**Authors:** Bangbei Wan, Ning Ma, Weiying Lu

**Affiliations:** 1 Reproductive Medical Center, Hainan Women and Children’s Medical Center, Haikou, Hainan, China; 2 Department of Urology, Central South University Xiangya School of Medicine Affiliated Haikou Hospital, Haikou, Hainan, China

**Keywords:** Bladder cancer, Body fat mass, Mendelian randomization, Genome-wide association study, Single-nucleotide polymorphisms

## Abstract

**Background:**

Observational studies have suggested that obesity is associated with the risk of bladder cancer (BCa). However, their causal relationship remains unclear. This study aimed to prove the causal relationship between obesity and the risk of BCa by using Mendelian randomization.

**Methods:**

Single-nucleotide polymorphisms (SNPs) correlated with body fat indexes were screened from several genome-wide association studies (GWAS) with more than 300,000 individuals. Summary-level genetic data of BCa-related GWAS were obtained from a European cohort with a sample size of 218,792. An inverse-variance-weighted (IVW) method was used as the major MR analysis. The MR-Egger regression, IVW regression, leave-one-out test, and MR-Pleiotropy Residual Sum and Outlier methods were used to test the reliability and stability of MR results.

**Results:**

Genetically predicted per 1-SD increase in body fat indexes (whole body fat mass, and the right leg, left leg, right arm, left arm, and trunk fat mass) were associated with increased BCa risk with values of 51.8%, 77.9%, 75.1%, 67.2%, 59.7%, and 36.6%, respectively. Sensitivity analyses suggested that the genetically determined risk effect of obesity on BCa was stable and reliable.

**Conclusions:**

Our study provided powerful evidence to support the causal hypothesis that the genetically predicted high body fat mass was associated with a risk increase for BCa. The finding is a new idea for drawing up prevention strategies for BCa.

## Introduction

Bladder cancer (BCa), a common urological malignant neoplasm, is primarily derived from malignant transitional epithelial cells (Lai et al., 2021). BCa is clinically divided into non-muscle-invasive and muscle-invasive BCa (Patel, Oh & Galsky, 2020). Non-muscle-invasive and muscle-invasive BCa represent approximately 70% and 30% of the newly diagnosed cases of BCa, respectively (Lai et al., 2021). Currently, radical cystectomy is a primary treatment used for patients with BCa. The quality of patients’ life is quite poor after surgical operations, and they are faced with a certain recurrent risk (Stein et al., 2001; Yafi et al., 2011). The new global BCa cases and deaths, were more than 550,000 and 200,000, respectively, in 2018 (Richters, Aben & Kiemeney, 2020). New cases and deaths from BCa were approximately 81,180 and 17,100, respectively, in the United States in 2022 (Siegel et al., 2022). Therefore, identifying the risk factors of BCa and taking appropriate interventions are critical to reducing BCa morbidity and mortality.

Obesity is a serious public health problem worldwide and the main causal factor for cancers (Avgerinos et al., 2019; Fonteneau et al., 2022; Hopkins, Goncalves & Cantley, 2016). Increasing evidence from observational studies suggests obesity is a key driver of BCa risk and progression and influences patient prognosis (Lin et al., 2018; Lu & Tao, 2021; Noguchi, Liss & Parsons, 2015; Qin et al., 2013). Based on observational research, being overweight was significantly correlated with the incidence of BCa (*r* ^2^ = 0.36) (Rezaei et al., 2019). The results from a meta-analysis included 15 cohort studies with more than 14,201,500 individuals, indicating a linear correlation between body mass index (BMI) and risk of BCa (Sun et al., 2015). BMI has been used as a primary indicator of obesity to evaluate the association of risk of BCa. This process could be a limitation and source of bias, because BMI does not reflect fat from lean mass and neglects fat distribution (Neeland, Poirier & Despres, 2018; Roberson et al., 2014). Hence, some new indicators need to be used to precisely re-assess the correlation between obesity and the risk of BCa.

Mendelian randomization (MR), an epidemiologic method that uses genetic variants as instrumental variables to estimate the causal association between exposure and outcome, is on a par with randomized controlled trial (RCT) on controlling residual confounding factors (Burgess, Butterworth & Thompson, 2013; Davies, Holmes & Davey Smith, 2018). In the present study, a two-sample MR was carried out to investigate the causal relationship between obesity-related indexes (*e.g.*, leg fat, arm fat, trunk fat, and whole body fat masses) and the risk of BCa. The inverse variance weighted (IVW) algorithm was used as the primary analysis approach to evaluate the potential causation. Obesity has an adverse effect on human health. Clarifying obesity’s potential causal effect on the risk of BCa is crucial for drawing up public policies to prevent BCa. To our knowledge, our study is the first to explore the causal relationship between obesity and the risk of BCa using an MR approach.

## Materials & Methods

### Study design and assumptions

**Figure 1:**
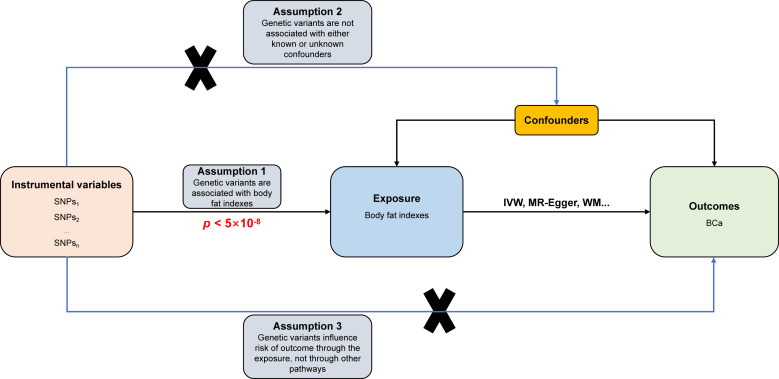
Directed acyclic flowchart of MR process for investigating the causal relationship between body fat indexes and the risk of BCa. Instrumental variable (IV) assumptions: (1) IVs must be strongly associated with body fat indexes (*p* < 5 ×10^−8^); (2) IVs must not be correlated with any unmeasured confounders of body fat indexes *versus* BCa relationship; (3) IVs should only affect the risk of BCa *via* body fat indexes. SNPs, single-nucleotide polymorphisms; BCa, bladder cancer; IVW, inverse variance weighted; WM, weighted median.

The study design and fundamental assumptions of an MR investigation are displayed in Fig. 1. The summary statistical data of genome-wide association studies (GWAS) were extracted from the IEU Open GWAS database (https://gwas.mrcieu.ac.uk/). The data include six body fat indexes’ datasets (bilateral legs fat, bilateral arms fat, trunk fat, and whole-body fat masses) and one dataset related to BCa. These datasets were used to conduct the two-sample MR investigation to explain the causal association between obesity and the risk of BCa.

### Data sources

The summary-level genetic data correlated with the six body fat indexes of European populations were obtained from the IEU Open GWAS database. These data from the UK Biobank cohort of the Neale lab and sample size information were as follows: leg fat mass (right) with 331,293 individuals, leg fat mass (left) with 331,275 individuals, arm fat mass (right) with 331,226 participants, arm fat mass (left) with 331,164 participants, trunk fat mass with 331,093 populations, and whole-body fat mass with 330,762 populations. Then, single nucleotide polymorphisms (SNPs) that are independently associated with the six body fat indexes were extracted and treated as instrumental variables according to these conditions: a genome-wide significance threshold (*p* < 5 ×10^−8^) and independence among SNPs in linkage disequilibrium (*r*^2^ < 0.001; clumping distance, 10,000 kb). The independence of SNPs was ensured using the PhenoScanner database (http://www.phenoscanner.medschl.cam.ac.uk/) to identify and remove SNPs that are associated with confounders or BCa. The instrumental variables’ power in MR analyses was assessed by calculating the F statistic based on the previously described methods (Pierce, Ahsan & Vanderweele, 2011). The GWAS summary-level data correlated with BCa from a FinnGen biobank cohort of European ancestry (1,115 cases and 217,677 controls) were downloaded using the IEU Open GWAS database. All participants in the six body fat indexes research programs were not screened for the BCa cohort.

### Statistical analysis

The causal relationship between obesity and the risk of BCa was investigated by conducting a univariable two-sample MR analysis. The IVW (Burgess, Butterworth & Thompson, 2013) algorithm was used as the principal causal effect evaluating method to calculate the pooled effect of all SNPs. The MR-Egger (Bowden, Davey Smith & Burgess, 2015), weighted median (Bowden et al., 2016), simple mode (Hartwig, Davey Smith & Bowden, 2017), and weighted mode (Hartwig, Davey Smith & Bowden, 2017) algorithms were used to assess the reliability and robustness of the results. The primary IVW and MR-Egger algorithms were used to examine the heterogeneity of SNPs. At *p* > 0.05, heterogeneity is considered absent in the included instrumental variables, and the effect of the heterogeneity on the estimation of causal effects can be ignored. In the presence of heterogeneity, the multiplicative random effects IVW model was then employed to estimate the effect size. The Egger regression algorithm and the MR-pleiotropy residual sum outlier (MR-PRESSO) (Verbanck et al., 2018) were used to inspect the potential bias from directional pleiotropy. Outliers were identified and removed using the MR-PRESSO method. The leave-one-out method was used to detect whether existing single SNPs affected the total effect of IVW. The directionality that obesity-related indexes cause BCa was confirmed *via* the MR Steiger test (Hemani, Tilling & Davey Smith, 2017), and *p* < 0.05 was considered statistically significant. All MR analyses were conducted using the TwoSampleMR and MRPRESSO packages in R software (version 4.1.2).

## Results

The causal relationship between body fat indexes and the risk of BCa was investigated using IVW as the primary method. All 261 independently available SNPs associated with whole-body fat mass were used to calculate the effect size of the genetically predicted whole-body fat mass on the risk of BCa. The results obtained using the IVW method displayed that the odds ratio (OR) was 1.518 (95% CI [1.128–2.042], *p* = 0.006). A total of 267 independently available SNPs related to leg fat mass (right) were used to compute the effect size of the genetically predicted leg fat mass (right) on the risk of BCa. The result from IVW method indicated that the OR was 1.779 (95% CI [1.246–2.540], *p* = 0.002). All 266 independent SNPs correlated with leg fat mass (left) were used to calculate the effect size of the genetically predicted leg fat mass (left) on the risk of BCa. The results obtained using the IVW method showed that the OR was 1.751 (95% CI [1.202–2.551], *p* = 0.004). A total of 255 independent SNPs related to arm fat mass (right) were used to compute the effect size of the genetically predicted arm fat mass (right) on the risk of BCa. The result obtained using the IVW method indicated that the OR was 1.672 (95% CI [1.247–2.243], *p* = 0.001). All 253 independently available SNPs related to arm fat mass (left) were used to estimate the effect size of the genetically predicted arm fat mass (left) on the risk of BCa. The result obtained from the IVW method exhibited an OR of 1.597 (95% CI [1.175–2.169], *p* = 0.003). A total of 270 independent SNPs related to trunk fat mass were used to calculate the effect size of the genetically predicted trunk fat mass on the risk of BCa. The result of IVW method indicated that the OR was 1.366 (95% CI [1.014–1.841], *p* = 0.040). The abovementioned results are summarized in Table 1. The F statistic of all available SNPs was greater than 10 in this MR, indicating no weak-instrument bias (Table 1).

**Table 1:** MR results of body fat indexes on the risk of BCa.

**Exposure**	**Method**	**No. of SNPs**	***p***	**OR (95% CI)**	***p***_−*het*_	***p*** **-**_*intercept*_	***p***_−*global*_	***p***_−*steiger*_	***F*** **statistic**
Whole body fat mass	MR Egger	261	0.966	1.020 (0.416–2.502)	0.377	0.358			
	Weighted median	261	0.004	1.931 (1.237–3.014)					
	**Inverse variance weighted**	261	0.006	1.518 (1.128–2.042)	0.379			0.000	94.983
	Simple mode	261	0.133	2.726 (0.740–10.047)					
	Weighted mode	261	0.043	2.495 (1.032–6.032)					
	MR-PRESSO (raw)	261	0.004	1.534 (1.243–1.824)			0.505		
Leg fat mass (right)	MR Egger	267	0.304	1.790 (0.592–5.416)	0.452	0.991			
	Weighted median	267	0.004	2.301 (1.305–4.059)					
	**Inverse variance weighted**	267	0.002	1.779 (1.246–2.540)	0.469			0.000	93.140
	Simple mode	267	0.457	1.813 (0.379–8.674)					
	Weighted mode	267	0.134	2.434 (0.764–7.751)					
	MR-PRESSO (raw)	267	0.001	1.799 (1.447–2.150)			0.573		
Leg fat mass (left)	MR Egger	266	0.197	2.126 (0.678–6.672)	0.152	0.725			
	Weighted median	266	0.005	2.434 (1.307–4.532)					
	**Inverse variance weighted**	266	0.004	1.751 (1.202–2.551)	0.161			0.000	94.237
	Simple mode	266	0.359	2.120 (0.427–10.512)					
	Weighted mode	266	0.111	2.491 (0.814–7.621)					
	MR-PRESSO (raw)	266	0.002	1.771 (1.404–2.138)			0.264		
Arm fat mass (right)	MR Egger	255	0.941	1.033 (0.436–2.446)	0.600	0.245			
	Weighted median	255	0.003	2.089 (1.283–3.399)					
	**Inverse variance weighted**	255	0.001	1.672 (1.247–2.243)	0.594			0.000	95.852
	Simple mode	255	0.106	2.868 (0.803–10.241)					
	Weighted mode	255	0.048	2.635 (1.014–6.843)					
	MR-PRESSO (raw)	255	0.000	1.680 (1.393–1.967)			0.688		
Arm fat mass (left)	MR Egger	253	0.736	1.166 (0.477–2.851)	0.180	0.464			
	Weighted median	253	0.001	2.230 (1.404–3.541)					
	**Inverse variance weighted**	253	0.003	1.597 (1.175–2.169)	0.186			0.000	96.172
	Simple mode	253	0.086	2.965 (0.863–10.190)					
	Weighted mode	253	0.024	2.709 (1.145–6.408)					
	MR-PRESSO (raw)	253	0.001	1.642 (1.340–1.945)			0.222		
Trunk fat mass	MR Egger	270	0.975	0.986 (0.395–2.460)	0.072	0.460			
	Weighted median	270	0.014	1.736 (1.116–2.700)					
	**Inverse variance weighted**	270	0.040	1.366 (1.014–1.841)	0.074			0.000	93.476
	Simple mode	270	0.153	2.506 (0.713–8.816)					
	Weighted mode	270	0.081	2.405 (0.901–6.418)					
	MR-PRESSO (raw)	270	0.033	1.375 (1.082–1.667)			0.116		

**Notes.**

BCa bladder cancer IVW inverse variance weighted OR odds ratio CI confidence intervals p-het *p* value for heterogeneity using Cochran *Q* test p-intercept *p* value for MR-Egger intercept MR-PRESSO Mendelian randomization-pleiotropy residual sum outlier p-global *p* value for MR-PRESSO global test p-steiger *p* value for MR-Steiger test SNP single-nucleotide polymorphism

**Figure 2:**
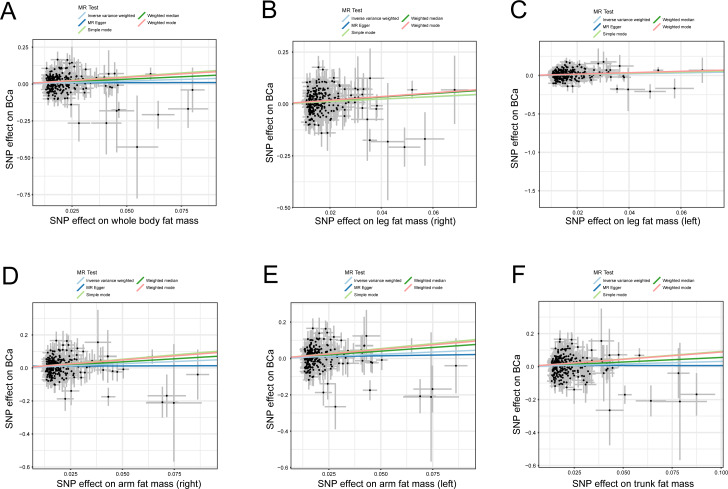
Scatter plots of body fat indexes with the risk of BCa. Scatter plot demonstrating the effect of each body fat indexes-associated SNP on the risk of BCa on the log-odds scale, (A) whole body fat mass, (B) leg fat mass (right), (C) leg fat mass (left), (D) arm fat mass (right), (E) arm fat mass (left), and (F) trunk fat mass; The slopes of each line represent the causal association for each method. MR, Mendelian randomization; SNP, single-nucleotide polymorphism; BCa, bladder cancer.

The credibility of the above results was determined using four approaches (MR-Egger, weighted median, simple mode, and weighted mode) to affirm the causal direction from body fat indexes to the risk of BCa. The evidence from statistical analysis supports that a genetically predicted increase in body fat indexes is a risk factor for BCa (Fig. 2).

Several sensitivity analyses were employed to assess the stability of the above results. The Cochran’s *Q* test in the fixed-effect IVW model (whole body fat mass: *p* = 0.379; leg fat mass (right): *p* = 0.469; leg fat mass (left): *p* = 0.161; arm fat mass (right): *p* = 0.594; arm fat mass (left): *p* = 0.186; trunk fat mass: *p* = 0.074) and MR Egger model (whole body fat mass: *p* = 0.377; leg fat mass (right): *p* = 0.452; leg fat mass (left): *p* = 0.152; arm fat mass (right): *p* = 0.600; arm fat mass (left): *p* = 0.180; trunk fat mass: *p* = 0.071) indicate the absence of heterogeneity in the instrumental variables in all results (Table 1). The statistical evidence from the MR-Egger intercepts and the MR-PRESSO global tests uniformly displayed no horizontal pleiotropy in the MR analysis (Table 1). In addition, the leave-one-out analysis indicated that no single SNP modified the combined estimate effect, suggesting the stability and credibility of our results (Fig. S1). The MR Steiger test proved the causal assumption of body fat indexes and the risk of BCa, and the results show that the influence of body fat indices on the risk of BCa was a correct causal direction (all *p* = 0.000; Table 1).

## Discussion

In this MR investigation, the most recent and most extensive GWASs data from European populations were used to investigate the causal relationship between body fat indexes and the risk of BCa. Results show that obesity was causally correlated with the risk of BCa. All statistical analyses consistently supported that our findings were steady and credible.

Obesity is a predisposing factor for various diseases and severely affects human health (Conway & Rene, 2004; Kolotkin, Meter & Williams, 2001; Park et al., 2014; Piche, Tchernof & Despres, 2020). A previous meta-analysis that included 1,2017 individuals showed that BMI was associated with the prognosis of BCa, and a higher BMI with a higher recurrence and a lower overall survival risk was obtained (Lin et al., 2018). The results from a recent observational study with a large sample revealed that overweight (*r* ^2^ = 0.36, *p* < 0.001) and obesity (*r* ^2^ = 0.34, *p* = 0.001) have a significant positive correlation with the incidence of BCa in Asian populations (Rezaei et al., 2019). A recent meta-analysis also reported that obesity is associated with the occurrence risk of BCa, and the risk ratio (RR) was 1.1 (95% CI [1.07–1.13]) (Shi et al., 2021). The result of dose analysis suggested that a 5 kg/m^2^ increase in BMI with a RR of 1.03 (95% CI [1.02–1.05]) in BCa (Shi et al., 2021). A large prospective study investigated the relationship between BMI and BCa and found that a higher BMI triggers BCa (Koebnick et al., 2008). A meta-analysis investigated modifiable risk factors of BCa and found that obesity was associated with BCa with RR of 1.10 (Al-Zalabani et al., 2016). By contrast, a recent observational research reported that overweight and obesity are negatively correlated with the incidence risk of BCa in Egyptian women after menopause. The ORs were 0.59 (95% CI [0.43–0.81]) and 0.26 (95% CI [0.18–0.38]) (Amr et al., 2019). However, the evidence of a meta-analysis showed that overweight and obesity are not associated with the incidence risk of BCa (Xue et al., 2017). A cohort study with a large sample revealed that obesity was not correlated with the incidence risk for BCa (Guo et al., 2014). Among the abovementioned studies, BMI was used as the primary indicator to measure obesity. However, BMI was criticized recently for not discriminating fat from lean mass and neglecting fat distribution (Bell et al., 2018; Trestini et al., 2018). A “U-shaped” relationship between BMI and diseases is frequently reported in previous studies (Kastorini & Panagiotakos, 2012; Lennon et al., 2016; Ryu et al., 2021; Sato et al., 2014; Trestini et al., 2018). Hence, reliable indicators need to be determined to reflect obesity correctly and thus accurately reveal the relationship between obesity and the risk of disease (Cespedes Feliciano, Kroenke & Caan, 2018; Park, Peterson & Colditz, 2018). Body fat indexes are better indicators than BMI for the measurement of obesity and assessment of the relationship between obesity and disease (Bell et al., 2018; Kesztyus, Lampl & Kesztyus, 2021; Reis et al., 2009). In the present study, six body fat indexes were used to evaluate the causality between obesity and the risk of BCa, and the results show that genetically determined obesity was a risk factor for BCa. Genetically predicted per 1-SD increase in body fat indexes (whole body fat mass and the right leg, left leg, right arm, left arm, and trunk fat mass) were associated with increased BCa risk with values of 51.8%, 77.9%, 75.1%, 67.2%, 59.7%, and 36.6%, respectively,). Based on the findings, the appropriate control of weight may effectively reduce the risk of BCa. This study first revealed the causal association between body fat indexes and the risk of BCa. It is beneficial to map out preventive strategies for BCa in clinical study.

Our study has some major strengths: First, it included the GWAS data derived from recent large sample studies. Moreover, the MR method allows us to investigate causality, thus avoiding confounding and reverse causation (Emanuelsson et al., 2019). In addition, it included SNPs, which were all strong instrumental variables. Multiple MR investigations also validated the causality between body fat indexes and the risk of BCa. Finally, multiple sensitivity analyses supported the causal inferences.

This study has some limitations. First, our study only was conducted in European populations, and whether it applies to non-European ancestry is unclear. Second, although body fat masses were positively correlated with the risk of BCa, a certain threshold was unknown about body fat masses, thus increasing the risk of BCa. Third, it is necessary to validate the finding by using other datasets with body fat mass. Finally, considering the data limitations, the effect of body fat masses on the risk of BCa between males and females was not investigated.

## Conclusions

In conclusion, the present study provided substantial evidence to support the causal hypothesis that the genetically predicted high body fat masses were associated with an increased risk of BCa. In addition, the genetically determined risk effect of body fat masses on BCa is lifelong. Therefore, our findings are significant in mapping out prevention strategies for BCa.

##  Supplemental Information

10.7717/peerj.14739/supp-1 Supplemental Information 1 Leave-one-out plots of body fat indexes with the risk of BCa (A) whole body fat mass, (B) leg fat mass (right), (C) leg fat mass (left), (D) arm fat mass (right), (E) arm fat mass (left), (F) trunk fat mass. Leave-one-out analysis for IVW MR of body fat indexes on BCa in summary-level analyses. SNP, single-nucleotide polymorphism; BCa, bladder cancer; MR, Mendelian randomization.

10.7717/peerj.14739/supp-2 Supplemental Information 2 STROBE-MR checklist, raw data, and R Code

## Additional Information and Declarations

### Data Availability

The following information was supplied regarding data availability: Raw data and code are available in the Supplemental Files.
