# Supplementary material for: Mendelian randomization investigation identified the causal relationship between body fat indexes and the risk of bladder cancer
Source: PeerJ. 2023 Jan 20;11:e14739. doi: 10.7717/peerj.14739 (PMC9869775; doi:10.7717/peerj.14739)
Supplement: Supplemental Information 1 — (A) whole body fat mass, (B) leg fat mass (right), (C) leg fat mass (left), (D) arm fat mass (right), (E) arm fat mass (left), (F) trunk fat mass. Leave-one-out analysis for IVW MR of body fat indexes on BCa in summary-level analyses. SNP, single-nucleotide polymorphism; BCa, bladder cancer; MR, Mendelian randomization. [file peerj-11-14739-s001.pdf]

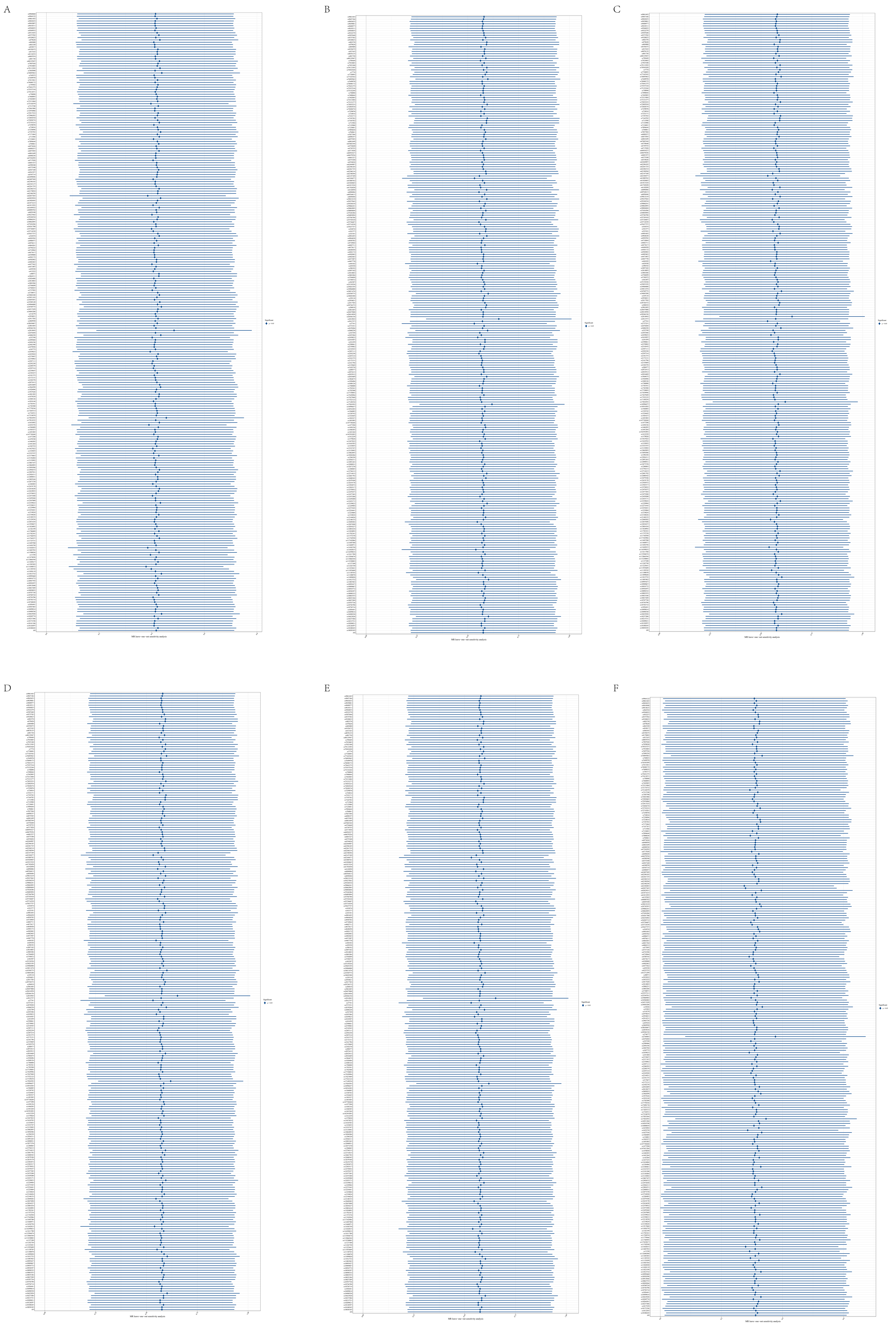
A
B
C
D
E
F
MR leave−one−out sensitivity analysis
Significant
p < 0.05
